# Variations of Scale Height at F-Region Peak Based on Ionosonde Measurements during Solar Maximum over the Crest of Equatorial Ionization Anomaly Region

**DOI:** 10.1155/2014/397402

**Published:** 2014-08-04

**Authors:** Yu-Jung Chuo

**Affiliations:** Department of Information Technology, Ling Tung University, Taichung 408, Taiwan

## Abstract

Scale height is an important parameter in characterizing the shape of the ionosphere and its physical processes. In this study, we attempt to examine and discuss the variation of scale height, *H*_*m*_, around the F-layer peak height during high solar activity at the northern crest of the equatorial ionization anomaly (EIA) region. *H*_*m*_ exhibits day-to-day variation and seasonal variation, with a greater average daily variation during daytime in summer. Furthermore, the diurnal variation of *H*_*m*_ exhibits an abnormal peak at presunrise during all the seasons, particularly in winter. This increase is also observed in the F2-layer peak height for the same duration with an upward movement associated with thermospheric wind toward the equator; this upward movement increases the N_2_/O ratio and *H*_*m*_, but it causes a decrease in the F2-layer maximum critical frequency during the presunrise period.

## 1. Introduction

Investigation of the ionospheric electron density profile *Ne*(*h*) is an important topic for many practical problems and contributes to our understanding of the structure of the ionosphere, ionospheric dynamics, and radio wave for telecommunications. The most important ionospheric region to investigate is the ionospheric F region, which is divided into three subregions: F1 and F2, which are considered the bottomside of the ionosphere, and the upper F region also known as the topside ionosphere. F1 is the lowest region and is dominated by photochemical processes, whereas the upper F region is dominated by diffusion. F2 is in between these two regions, where there is a transition from chemical to diffusion dominance. Examining variations in the ionosphere's shape will help us to study the physical processes in the ionosphere.

The ionospheric bottomside electron density profile was derived from ground-based observations using an ionosonde, digisonde, and incoherent scatter radar in the form of ionograms. The ionospheric topside electron density profile can be obtained from topside sounder measurements or by theorematic models. Recently, many analytical functions and empirical modeling techniques have been reported to study the topside ionosphere, such as Chapman's function and exponential, parabolic, and Epstein functions [1–8]. Reinisch and Huang [5] used scale heights derived from ionograms recorded at Millstone Hill (a middle latitude site) and Jicamarca (located at the geomagnetic equator) stations for modeling the topside ionospheric profile. They proposed a convenient method to derive the topside ionospheric profile based on the Chapman scale height, *H*_*m*_, around the F2-layer peak height. They have shown that variations in scale height derived using the *α*-Chapman function above the F2-layer peak are very small and have assumed a constant scale height for the interpolation of the topside ionosphere.

Over the past few years, many studies have been conducted on this subject, like that of [9–15]. Tulasi Ram et al. [14] studied *H*_*m*_ around the F2-layer at all 13 stations and have shown maximum values in summer during daytime. Furthermore, Zhang et al. [10] also studied the *H*_*m*_ at the F-region peak height over Hainan (19.4°N, 109°E) and showed two conspicuous peaks occurring at local noon and presunrise. Their results show a high correlation between *H*_*m*_ and *B*0 at different latitudes and equivalent slab thicknesses, *τ*, at low latitudes. In addition, Lee and Reinisch [12] presented a postsunset peak in *H*_*m*_ during the equinox and summer at the equatorial region, Jicamarca (12.0°S, 76.9°W), during high solar activity. Although numerous studies of scale height have been carried out in many locations, it has not yet been examined at the northern crest of the equatorial ionization anomaly (EIA) area.

Since *H*_*m*_ plays an important role in the ionospheric vertical profile, especially for deriving and studying the topside ionosphere, we have analyzed the diurnal and seasonal variations of *H*_*m*_ in order to study the ionospheric dynamics in the crest of the EIA area. In this work, we examine the ionograms recorded in 1999 at Chung-Li (24.9°N, 121.1°E) to derive *H*_*m*_ during high solar activity. Furthermore, this investigation also studies the correlation between *H*_*m*_, F2-layer peak height (*hmF*2), *B*0, and *τ* to investigate ionospheric physics processes over the crest of the EIA in East Asia.

## 2. Material and Methods

In our study, we collected data on the F2-layer critical frequency (*foF*2), *hmF*2, *B*0, *H*_*m*_, and the total electron content (TEC) from the Chung-Li ionosonde station (24.9°N, 121.1°E) and YMSM GPS receiver (25.2°N, 121.6°E). We processed the ionogram traces manually and transferred them to computers, capturing the ionospheric true height profiles for more than 34,000 ionograms taken in 1999 during high solar activity. These data are classified into three periods: equinox (March, April, September, and October), summer (May–August), and winter (November–February).

The true height plasma frequency profile is calculated using the standard true-height inversion POLynomial ANalysis program (POLAN) [15]. POLAN is widely accepted for inversion of ionograms obtained by classical ionosonde [16] and allowed our measurements of *foF*2, *hmF*2, *B*0, and *H*_*m*_. This study processes a given set of virtual-height data from ionograms with a manual work. Results obtained by POLAN are therefore normally stored by arrays giving the scaled frequencies and corresponding real heights. The ionograms were recorded every 15 minutes at Chung-Li station. GPS TEC data are used to calculate *τ*, which is defined as the ratio of TEC/NmF2.

The goal of this paper is focused on the day-to-day and seasonal variations in *H*_*m*_ and the correlation between *H*_*m*_ and other ionospheric parameters such as *hmF*2, *B*0, and *τ*. In addition, we tried to get a clearer picture of ionospheric dynamics in the northern crest of EIA area.

## 3. Results and Discussion

**Figure 1:**
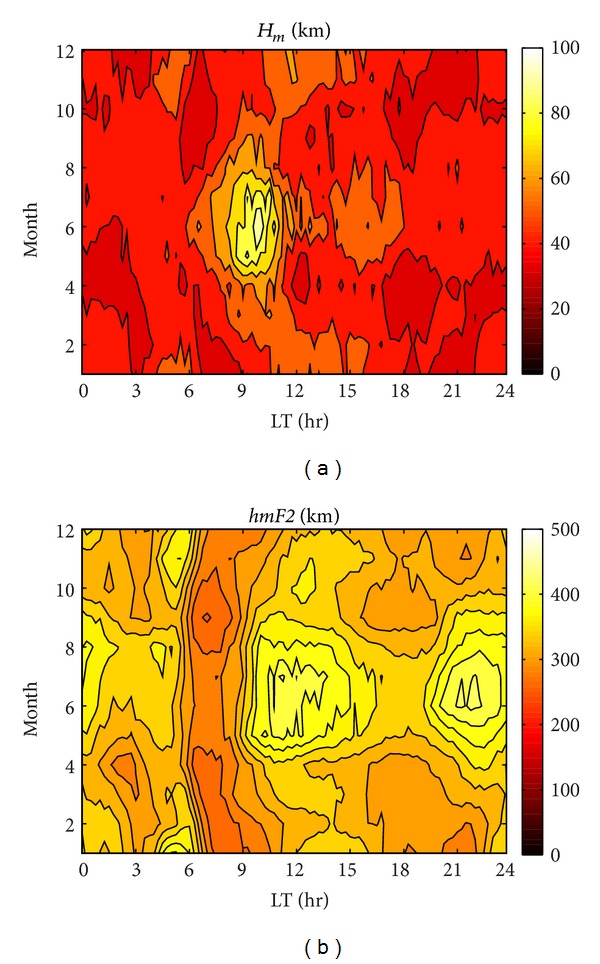
Contour illustrations of *H*_*m*_ (a) and *hmF*2 (b) during 1999.

In order to obtain average diurnal variations in *foF*2, *hmF*2, *B*0, *H*_*m*_, and *τ* values every month during the whole year, we calculate median values for all the days in each month in 1999 during periods of high solar activity and smooth out the data. These results are illustrated in Figure 1 and represent seasonal variations in *H*_*m*_, with a maximum appearing during daytime in June and August; lower values appear during nighttime in the winter months (Figure 1(a)). In addition, the daytime peak of *H*_*m*_ also shows a seasonal variation; during the equinox and summer, the peak appears during 0800–1030 LT and, in winter, the peak appears later around 1030–1500 LT.  Figure 1(b) shows the contour plot in *hmF*2, which has an apparent higher value during 1000–1300 LT in summer (May–August). Moreover, another conspicuous peak was observed around 2200 LT in summer. The summer daytime peak in *H*_*m*_ occurs at an earlier time than *hmF*2 since the increase in *H*_*m*_ could be associated with the sunrise. That is to say, electron density begins to increase rapidly owing to photoionization at sunrise and then rises slowly during the day [17]. It is known that the [H^+^]/[O^+^] ratio is very important at the F2 peak. After sunrise, the meridional neutral wind blows toward the pole and produces a diffusion effect from the topside ionosphere to lower altitudes. Furthermore, the bottomside ionospheric electron density still increases from photoionization during daytime, which is associated with the increase in [O^+^] and uplift of the F2 layer to a higher altitudes, and it reduces the loss rate and thereby still increases the electron content around the F2 peak.

**Figure 2:**
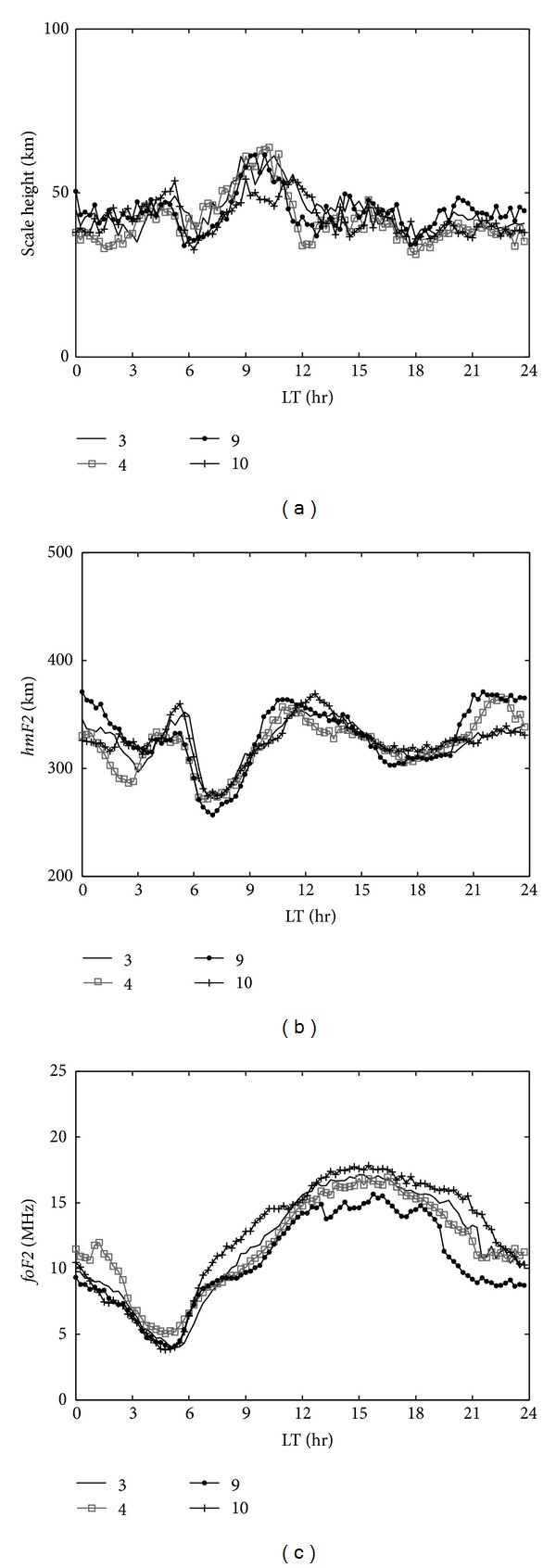
*H*_*m*_ (a), *hmF*2 (b), and *foF*2 (c) parameters for March (solid line), April (gray rectangle line), September (dotted line), and October (cross line), that is, the equinoctial months.

Figures 2(a)–2(c) show the monthly median values of *H*_*m*_, *hmF*2, and *foF*2 in the F2 region for equinoctial months: March (solid line), April (gray rectangle line), September (dotted line), and October (plus line) in 1999.  Figure 2(a) represents the diurnal variation in *H*_*m*_; daytime values were greater than nighttime values. In addition, two apparent peak values occur at about 0500 and 0900–1100 LT. The first *H*_*m*_ peak appears around 0400–0500 LT and increases to about 56 km and then falls rapidly to a trough value of approximately 38 km. The second *H*_*m*_ peak appears during 0900–1100 LT at 60–65 km and then decreases gradually.

Figure 2(b) shows the variation in *hmF*2 during equinoctial months. In general, there are three peaks to be observed: presunrise, local noon, and postsunset during the equinox, but the postdusk peak is not obvious in March and October.  Figure 2(c) shows a diurnal variation in *foF*2 and a peak and trough occurring during 1300–1600 LT and around 0500 LT, respectively, for all seasons. Comprehensively, the peak *H*_*m*_ during the equinox, which appears at 0400–0500 LT, contrasts with the increase in *hmF*2 during presunrise period while *foF*2 decreases to its minimum during the same period. This result indicates that the increase in *H*_*m*_ is associated with the equatorward neutral wind and leads to an increase in *hmF*2 and in the N_2_/O ratio and also causes a decrease in *foF*2 [18]. In addition, the shape of ionosphere is very sensitive to variations in the ratio of [H^+^]/[O^+^] at the F2 peak [19]. Moreover, the O^+^ density decreases with altitude [17]. Hence, the enhanced *hmF*2 decreases [O^+^] which leads to an increase in the [H^+^]/[O^+^] ratio and increases *H*_*m*_ at the F2 peak. The main peak in *H*_*m*_ starts increasing at 0600 LT and reaches its maximum value (~65 km) at about 1000 LT. During the same time period, *hmF*2 also increases from the trough (~250 km) to its maximum (~380 km) and then decreases gradually.

Fejer et al. [20] studied equatorial ionospheric vertical plasma drifts and showed a vertical upward plasma drift from 0600 to 1000 LT, and then the drift turned downward. The results seem to correspond to the variations in *H*_*m*_ at the EIA, which indicates the effect of dynamo electric fields during daytime. The slight increase in *H*_*m*_ during 1400–1600 LT is associated with the increase in plasma and decrease in *hmF*2 (Figures 2(b) and 2(c)).

**Figure 3:**
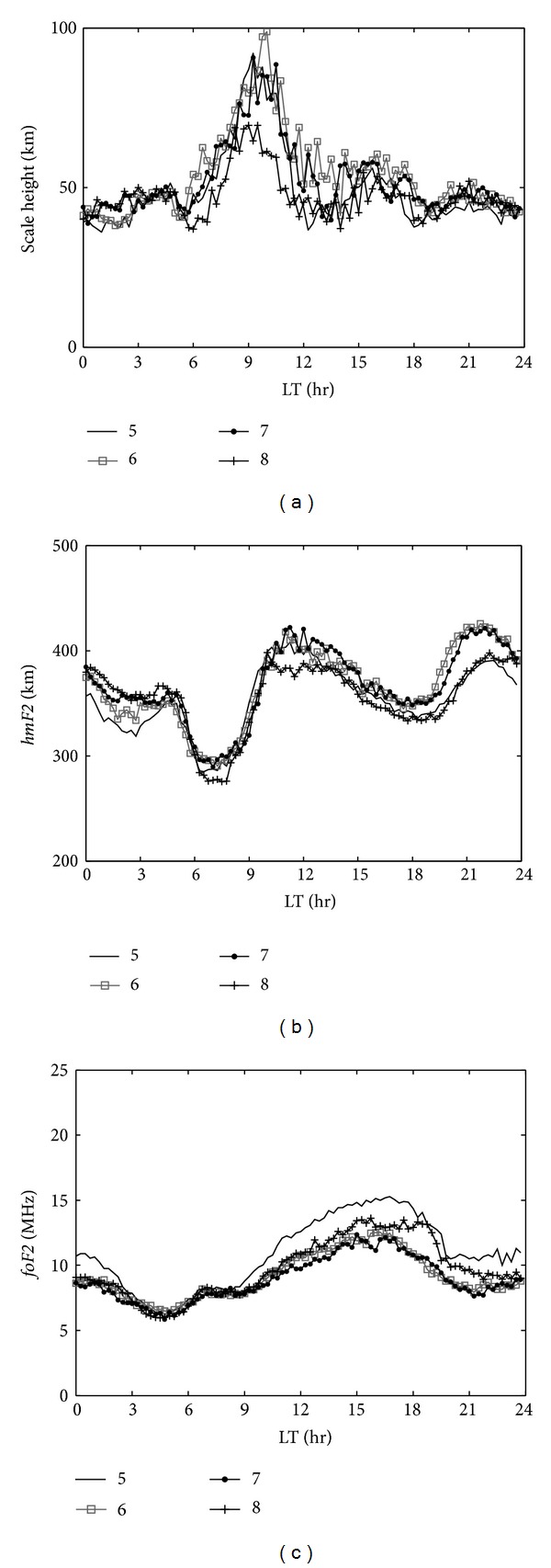
*H*_*m*_ (a), *hmF*2 (b), and *foF*2 (c) parameters for May (solid line), June (gray rectangle line), July (dotted line), and August (cross line), that is, the summer months.

**Figure 4:**
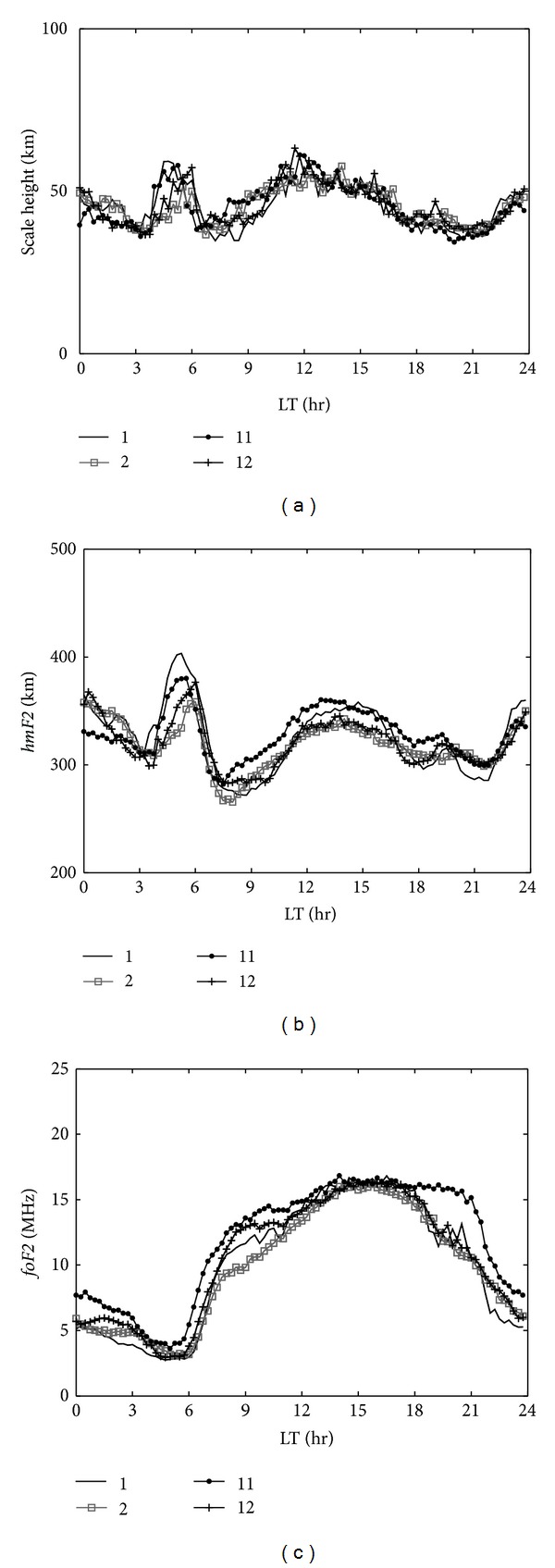
*H*_*m*_ (a), *hmF*2 (b), and *foF*2 (c) parameters for January (solid line), February (gray rectangle line), November (dotted line), and December (cross line), that is, the winter months.

Figures 3 and 4 show the same data as Figure 2, but for the summer and winter months. During summer, *H*_*m*_ shows a diurnal variation with the main peak appearing during 0900–1000 LT (Figure 3(a)). The main peak in *H*_*m*_ is higher in June (~100 km) and lower in August (~70 km).  Figure 3(b) shows the variation in *hmF*2 in summer and three peak values to be observed at presunrise, local noontime, and postsunset periods. Obviously, the peak value in *H*_*m*_ does not correspond to *hmF*2 during the daytime period. The variation in *H*_*m*_ is also attributed to the vertical plasma drifts induced by dynamoelectric fields [17]. In addition, a slight presunrise peak in *H*_*m*_ is observed in summer. This increase in *H*_*m*_ contrasted with a slight uplift in *hmF*2 during the presunrise period. A slight increase in *H*_*m*_ during 1500–1600 LT is concerned with the increase in *foF*2 (Figure 3(c)). Furthermore, a slight peak in *H*_*m*_ is observed during 1800–2100 LT and is also associated with an increase of *hmF*2, which is attributed to the equatorial prereversal enhancement caused by the occurrence of a strong postsunset eastward electric field [21].

In winter months, *H*_*m*_ shows two apparent peak values during presunrise and local noontime periods, with a maximum value of about 60 km at noon (Figure 4(a)). The main peak in *H*_*m*_ starts to increase from 40 km at 0600 LT and reaches its maximum value (~60 km) at 1200 LT, and *foF*2 also increases simultaneously and lasts until 1600 LT (Figure 4(c)). The variation in *H*_*m*_ also shows a similar trend that compares with the results of Fejer et al. [20], who studied the global vertical plasma drift during daytime at the equator. The presunrise peak in *H*_*m*_ is attributed to an uplift *hmF*2 (Figure 4(b)). If *H*_*m*_ is correlated to classic scale height, *H*, the temperature should be tightly correlated with *H*_*m*_.

**Figure 5:**
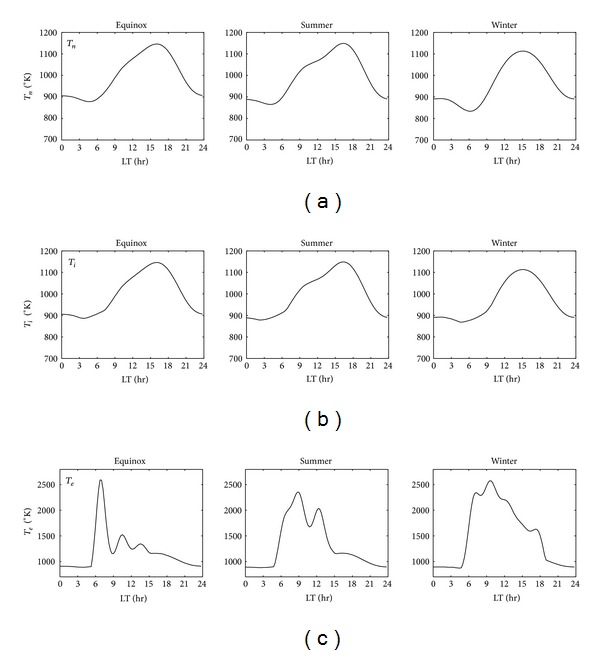
The variation in neutral gas (a), ion (b), and electron (c) temperature during the equinox, summer, and winter seasons.

Figure 5 illustrates the variations in temperature of neutral gas, ions, and electrons (*T*_*n*_, *T*_*i*_, and *T*_*e*_, resp.) and is measured using an IRI-2007 model over the Chung-Li location during 1999.  Figure 5(a) illustrates the variations in *T*_*n*_ during the equinox, summer, and winter. It shows a trough at 0500 LT and then increases to a maximum at around 1600 LT for all three seasons. It corresponds highly with the variations in *foF*2 and minimally with *H*_*m*_ during the daytime period. For the *T*_*i*_ (Figure 5(b)), the results represent a tendency similar to *T*_*n*_ for all three seasons.  Figure 5(c) shows *T*_*e*_ variations during different seasons. An abrupt increase can be seen from about 800°K to 2600°K during 0500–0700 LT and then a rapid decrease to 1200°K during the equinox. These results indicate that the temperature of neutral gas had stronger correlation with *foF*2 and weaker correlation with *H*_*m*_.

**Figure 6:**
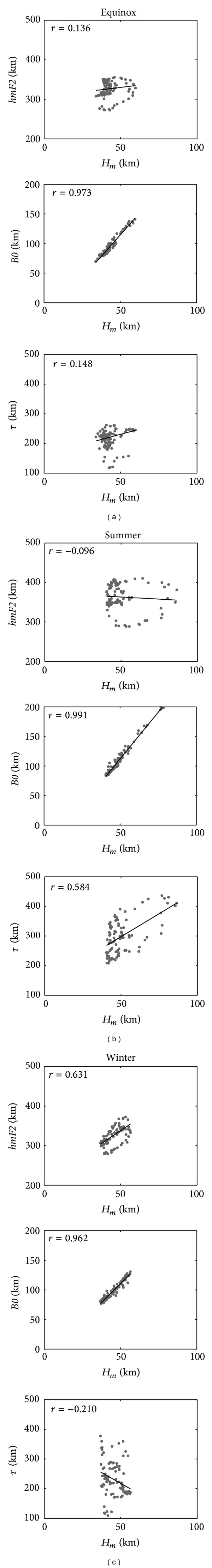
Correlation between *H*_*m*_ and *hmF*2, *B*0, and *τ* during the equinox (a), summer (b), and winter (c) seasons. The correlation coefficient *r* is also shown.

In addition, we also examined the correlation between *H*_*m*_ and ionospheric profile parameters such as ionospheric bottomside shape, *B*0, in the IRI model and the ionospheric equivalent slab thickness, *τ*.  Figure 6 shows the linear regression analysis between *H*_*m*_ and *hmF*2, *B*0, and *τ*. The top panels show the linear correlation between *H*_*m*_ and *hmF*2. The result shows a weak correlation (*r* varies in the range of −0.096 to 0.136) between *H*_*m*_ and *hmF*2, except for a middle correlation (*r* = 0.631) in winter. Furthermore, the resulting *H*_*m*_ compares with the result of vertical plasma drift during daytime at the equator reported by Fejer et al. [20] and finds that trends were similar during daytime. Our results indicate that *H*_*m*_ in the EIA area was strongly dominated by the **E** × **B** drift at the equator. The medium panel shows the correlation analysis between *H*_*m*_ and *B*0 during three seasons and represents a high correlation coefficient (*r* varies in the range 0.962–0.991) during the high solar activity period. This result is in agreement with Liu et al. [9] who indicated that *H*_*m*_ and *B*0 have a strong correlation at all times over Wuhan and over 13 other stations.

Further, Lee and Reinisch [12] studied *H*_*m*_ variations at an equatorial station, Jicamarca, and also indicated that *H*_*m*_ and *B*0 are highly correlated. Therefore, these results indicate that *H*_*m*_ values can be estimated using *B*0 values, which also corresponds to previous results at mid- and equatorial latitudes [9, 10, 12, 13]. Meanwhile, a study [22] indicated a strong correlation between *H*_*m*_ and *τ* at Millstone Hill. Zhang et al. [10] studied the variation of *H*_*m*_ at Hainan (19.4°N, 109.0°E) and also proposed a high correlation between *H*_*m*_ and *τ* during 2002–2004. Therefore, we also examined the comparison between *H*_*m*_ and *τ*. The seasonal values of *H*_*m*_ show a similar trend to *τ* over Chung-Li during high solar activity periods.  Figure 6 (bottom panel) shows the correlation analysis between *H*_*m*_ and *τ* during all seasons and represents a moderate correlation (*r* = 0.584) in summer and a weak or negative correlation during equinox (*r* = 0.148) and winter (*r* = −0.210). This result differs from the studies at other latitudes, mentioned above, since the EIA is more complex in its contribution to the electron density in the topside ionosphere, which is affected by electric fields and thermospheric winds. Therefore, the correlation was weak between *H*_*m*_ and *τ*.

## 4. Conclusions

This paper presents the diurnal and seasonal variations in Chapman scale height *H*_*m*_ around the F2-layer peak height at the northern crest of the EIA region, over the Chung-Li area station in Taiwan, during periods of high solar activity in 1999. *H*_*m*_, *hmF*2, *foF*2, and *B*0 values were derived from electron density profiles, which were calculated using the POLAN program. The key results obtained are as follows.

1. During the periods of high solar activity, the diurnal variations in *H*_*m*_ are generally characterized by higher daytime values as compared to the nighttime values in all the three seasons. Furthermore, monthly median daytime values of *H*_*m*_ are highest in summer and lowest in winter.
2. A presunrise peak in *H*_*m*_ is observed in all three seasons during high solar activity, particularly in the winter. This phenomenon is also observed in *hmF*2, whose increase is also more prominent during winter. These results are also observed at midlatitudes [10, 13] and equatorial latitudes [12]. Meanwhile, the presunrise peak in *H*_*m*_ is attributed to the uplift *hmF*2, which was caused by the zonal neutral wind. Murthy et al. [23] have mentioned that the meridian neutral wind turns southward at 0300 LT and reverse at 0500 LT for low latitudes. Hence, the F2 layer rises to higher altitude and causes a decrease in *foF*2 such that there is an increase in the ratio of N_2_/O.
3. During the day, *H*_*m*_ is highest in summer and strongly dominated by the vertical plasma drift produced at the equator. Further, our results show a poor correlation between *H*_*m*_ and *hmF*2, except for a middle correlation in winter. This vertical plasma drift due to a strong eastward electric field at the equator leads to plasma uplift and then diffuses down the magnetic field lines to the EIA region and causes an increase in the thickness (*τ*) of the topside ionosphere and *H*_*m*_.
4. During the postsunset period, a slight increase in *H*_*m*_ is observed in summer for high solar activity periods and is associated with an increase in *hmF*2 caused by prereversal enhancement at the equator.
5. A strong correlation is found between *H*_*m*_ and *B*0 values for all three seasons (*r* varies in the range 0.962–0.991). This result is also observed at equatorial latitudes and midlatitudes and suggests that *H*_*m*_ could be estimated by *B*0 [9, 12, 13]. In addition, *H*_*m*_ shows a poor correlation with *τ*, which differs from the results of Zhang et al. [10], who showed a strong correlation in a low-latitude station in Hai-nan.
6. This study provides more information about the ionospheric shape parameters during the solar maximum period over the crest of EIA region. The *H*_*m*_ parameter could be provided to compare with the ionospheric topside model and the radio occultation measurements on satellites. Besides, the bottomside ionospheric slab thickness, *B*0, could be estimated by the *H*_*m*_ to be easy in measuring *B*0 values. Furthermore, the *H*_*m*_ provides the information of ionospheric photoionization reactions and the ionospheric dynamics over the EIA region.
